# Trachoma; Its Importance as a Public Health Question

**Published:** 1918-10

**Authors:** A. B. Crain

**Affiliations:** Belton, Texas


### Trachoma; Its Importance as a Public Health Question.

A. B. CRAIN, M. D., BELTON, TEXAS.

Trachoma is a very old disease, probably as old as the Bible itself. It is also called by some "Sore Eyes," "Granulated Lids," etc., and is communicable from one person to another. Those who have it can in most cases be cured, and those who have not yet caught it can avoid doing so if they will learn how to protect themselves against infection. If every one, men, women and children, will learn about trachoma and take means for its prevention, the disease will in time disappear.

A large percentage of those who have trachoma and do not have it treated will eventually become blind. Even if blindness does not follow, every case of the disease causes some damage to the eyesight of those afflicted. Even without treatment the disease appears to get better, and the eye feels so much improved that the patient imagines he is cured, but sooner or later it will start up again even worse than before. Each of these attacks leaves its mark on the eyeball and affects the sight.

The sooner trachoma is treated and cured the less will be the injury to the sight. The longer it lasts the more damage it will also do to the eyelids; on this account, and because the eyelids will turn in and constantly lash the eyeball, pain and irritation continue and the sight will slowly but surely become worse until finally blindness results.

Trachoma is catching; not in the same way that measles, scarlet fever, and many other ailments are, but it is always due to some pus from a "Sore Eye" or trachomatous eye getting into a healthy eye. This can occur in many ways, such as the use of common basins, hankerchiefs, bed clothes, and I believe most frequently of all ways, by the use of the common face towel. I have seen in many places a long roller towel that is used by the entire household and all visitors, and in many homes it hangs on its roller until it is grimy from over use on many dirty faces.

Children at school may convey the disease to others by exchanging or using the same books, pencils, slates, clothes, or in playing games by blindfolding with cloths or fingers.

Also as the disease feels like a cinder in the eye, a friend may try to remove it and neglect to wash his hands before rubbing his own eyes.

In fact, the disease can be contracted in any manner which serves as a means of conveying infected discharges from a diseased to a healthy eye.

Any of you that has seen many cases of blindness, near blindness, all eye lashes gone, lids turned in and pus crusted around the eye, knows, that these cases are a pitiful, and I might say, repulsive sight; therefore I think that it is high time that we doctors, and health officers try to avail ourselves of any and all opportunities to help those unfortunates, and what is more important, keep others from getting into this shape.

So far as I know, there has been very little preventive or curative work done about trachoma in Texas, but I believe that a great deal of good can be accomplished along that line, if we will only take the proper amount of interest in the disease ourselves and in addition, interest our City Councils, School Boards, County Commissioners, School teachers and the general public, by telling and showing them the danger of this absolutely preventable disease.

When we get the public educated up to dangers and the ways of preventing a disease, they will demand that we use every precaution to prevent it.

In Bell County I have had the trachoma question to puzzle me from a Health Officer's standpoint for several years. For instance in some schools the teacher or some patron of the school would say to me that, "so and so's children have the sore eyes and we want you to stop them from school." I knew perfectly well that they should be stopped, but I also knew that upon examination of all the rest of the children in that school I would find a good many more that were, nearly, if not equally, as dangerous to the uninfected as the ones complained of, so to be fair, and at the same time to handle the question properly, has been very difficult for me.

I have on several occasions consulted the State Health Officer regarding the best way to handle trachoma, and I also wrote the United States Public Health Service, and fortunately, a few months ago the United States Public Health Service sent Surgeon John McMullen to Texas to make a survey of trachoma. I suppose because I had consulted him several times about trachoma, Dr. Collins, State Health Officer, sent Surgeon McMullen up to see Bell County first, for which I was very glad as his visit was a great benefit to me and I know it was to at least one hundred cases of trachoma, which he was kind enough to treat while there. He operated on sixty-five cases at a clinic, which was held in Belton one day. Since that time I have used his method and operated on twenty-five or thirty cases with excellent results.

When Dr. McMullen came and wanted to make a survey of the County I was glad of the chance to learn his methods of limiting trachoma, because I knew there were many cases in the County.

The survey included one thousand and two hundred (1200) rural school children from different points in the County. The percentage of trachoma in some schools was very low, while in others it was very high.

The average for the whole one thousand and two hundred (1200) children was 13 per cent.

We made a survey of only one town in the County and that was Belton. All white school children were examined, something over one thousand (1000) and we found 7 per cent. of them infected.

The majority of the cases in the schol were comparatively new cases; that is, they were not of the old, long-standing type. They were of the type that it is much easier to cure and that, I think, is one reason we had such remarkable results from the treatment.

After we made the city survey we began to devise ways and means of getting rid of as much of the trouble as we possibly could. Dr. McMullen agreed if I could arrange it to hold a clinic on one day, he would operate on all that I could get to come.

I took the matter up with the Mayor and School Board and they were heartily in favor of my plan, which was as follows: I had the School Superintendent send a letter to the parents of all infected children, telling them that their child had trachoma and by order of City Health Officer, could not come back to school until it was cured, and in the same letter he explained that they could all avail themselves of the proper treatment on a specified date.

I also had the support of all the local doctors and the ladies of the Red Cross Society. On the day of the clinic the ladies acted as nurses, and the doctors gave the anesthetics. By the efficient co-operation of all we were able to get the parents to see the wisdom of such a movement and practically every infected child that we had examined appeared for the treatment on the day set.

By this co-operation of the local doctors and Red Cross ladies and the Belton Sanitarium we were able to operate on sixty-five children in one day, and sixty-four took a general anesthetic.

This, I think, was the first trachoma clinic ever held in Texas, and Dr. McMullen, who had been in the work for ten or fifteen years, said it was the largest he ever held in one day.

The operation as performed by Dr. McMullen of the United States Public Health Service, was, briefly, as follows: First, the instruments used are a specially devised forceps for entering the eyelids, two small scalpels, horn spoon for protection of the cornea, Desmarres forceps, tooth brush, bichloride solution one to two thousand, and plain sterile gauze.

After everting the lids, the lid is scarified and the granules incised superficially with the scalpels, the surfaces gone over with the brush and the bichloride solution followed by thorough use of the gauze. The operation can be done with local anesthetic, but in young children and very nervous patients a general anesthetic will be required, because you can do a more thorough operation.

Immediately after the operation the conjunctive is washed with boric acid solution, with the eyelids everted in order to remove all blood clots and thus lessen the tendency to adhesions. When a radical grattage has been done, the eye should be under the surveilance of the surgeon during the twenty-four to forty-eight hours succeeding the operation to prevent the occurrence of adhesions. After-treatment consists of cleansing the eye every three hours with boric acid solution, followed by the use of 20 per cent. argyrol solution.

After five or six days, if uneven granules or rough surfaces are present, the lids should be everted and brushed lightly with a 2 per cent. silver nitrate solution, this to be repeated as often as necessary and as the individual case will stand.

The operation employed is a form of grattage, but the amount of traumatism depends entirely upon the individual case in hand, and what would be quite sufficient in one case would be inadequate in another.

The aim is to remove the abnormal tissue from the entire conjunctive and the operation is to be discontinued immediately after this has been accomplished. With the eyelid fully everted this can be determined by the smoothness of the surface and the appearance of the small blood vessels.

Some form of grattage had been practiced in the treatment of trachoma since the time of Hippocrates, but upon the quality and quantity of this operation depend the results.

I have followed up as closely as possible the cases in Belton on whom the operation was performed and to me the results have been remarkable. Less than 10 per cent. of the cases have needed further treatment; 75 per cnt of them were back in school in one week and all but two or three were back in 10 days.

So far as the City of Belton is concerned, I am going to inspect the schools next fall, when school begins, and make all infected persons be treated, if they are not financially able to have their specialist or family physician to treat them, I am going to do it for them.

In this way I hope to be able to keep trachoma practically out of our schools.

When it comes to a large county it will be more difficult to handle, because one must have the co-operation of the different School Boards, and the Commissioners Court would have to provide funds for the proper treatment of a large percentage of the county cases.

I hope that a better method will some time in the near future be available to infected patients in this State, I mean by this that I hope the United States Public Health Officer will establish trachoma hospitals in the regions of Texas where there are a large number of cases, as they have done, and are doing, in Tennessee, Kentucky, Florida and other states. I am sure that if the Health Officers of the State will take more interest in trachoma and will make investigations to find out how much is in their communities and will tell the United States Health Officer that we need their help, it will not be long in coming.

In the meantime we should be working for some concerted action as well as Federal aid; we, as individual Health Officers and Physicians, can do a great deal to control the disease by treating all of it that we can, and by teaching the public how to avoid infection.

It doesn't take an eye specialist to treat trachoma, in fact most of them I think, had rather not fool with it.

Any competent doctor can learn, in a very few minutes, how to operate by the method outlined in this paper; it is very simple and I know from experience and observing about one hundred cases that it is by far the most successful treatment I have yet seen.

I have merely stated to you, how I have tried to deal with it locally and personally and I will appreciate a liberal discussion and criticism of the method I have outlined for its partial control. However I know that the proper thing to do would be to establish Trachoma Hospitals throughout the infected parts of Texas and treat and cure all cases old and young. Some day I am sure this will be done. But the point I want to make is that we can do a great deal to lessen the disease by cleaning up our schools as I have tried to do in Belton. I know that a great many cases will become reinfected at home by father, mother, brother or sister, but when you treat one case in a family and they see the improvement, frequently the other members will also apply for treatment.

